# The Intake of Dicarbonyls and Advanced Glycation Endproducts as Part of the Habitual Diet Is Not Associated with Intestinal Inflammation in Inflammatory Bowel Disease and Irritable Bowel Syndrome Patients

**DOI:** 10.3390/nu15010083

**Published:** 2022-12-24

**Authors:** Marlijne C. G. de Graaf, Jean L. J. M. Scheijen, Corinne E. G. M. Spooren, Zlatan Mujagic, Marieke J. Pierik, Edith J. M. Feskens, Daniel Keszthelyi, Casper G. Schalkwijk, Daisy M. A. E. Jonkers

**Affiliations:** 1 Division Gastroenterology-Hepatology, Department of Internal Medicine, Maastricht University Medical Center+, 6229 ER Maastricht, The Netherlands; 2 NUTRIM School of Nutrition and Translational Research in Metabolism, Faculty of Health, Medicine and Life Sciences, Maastricht University, 6229 ER Maastricht, The Netherlands; 3 Department of Internal Medicine, Maastricht University Medical Center+, 6229 ER Maastricht, The Netherlands; 4 CARIM School for Cardiovascular Diseases, Faculty of Health, Medicine and Life Sciences, Maastricht University, 6229 ER Maastricht, The Netherlands; 5 Division of Human Nutrition and Health, Wageningen University & Research, 6708 WE Wageningen, The Netherlands

**Keywords:** inflammatory bowel disease, irritable bowel syndrome, dietary dicarbonyls, dietary advanced glycation endproducts, intestinal inflammation, faecal calprotectin

## Abstract

A Western diet comprises high levels of dicarbonyls and advanced glycation endproducts (AGEs), which may contribute to flares and symptoms in inflammatory bowel disease (IBD) and irritable bowel syndrome (IBS). We therefore investigated the intake of dietary dicarbonyls and AGEs in IBD and IBS patients as part of the habitual diet, and their association with intestinal inflammation. Food frequency questionnaires from 238 IBD, 261 IBS as well as 195 healthy control (HC) subjects were used to calculate the intake of dicarbonyls methylglyoxal, glyoxal, and 3-deoxyglucosone, and of the AGEs Nε-(carboxymethyl)lysine, Nε-(1-carboxyethyl)lysine and methylglyoxal-derived hydroimidazolone-1. Intestinal inflammation was assessed using faecal calprotectin. The absolute dietary intake of all dicarbonyls and AGEs was higher in IBD and HC as compared to IBS (all *p* < 0.05). However, after energy-adjustment, only glyoxal was lower in IBD versus IBS and HC (*p* < 0.05). Faecal calprotectin was not significantly associated with dietary dicarbonyls and AGEs in either of the subgroups. The absolute intake of methylglyoxal was significantly higher in patients with low (<15 μg/g) compared to moderate calprotectin levels (15–<50 μg/g, *p* = 0.031). The concentrations of dietary dicarbonyls and AGEs generally present in the diet of Dutch patients with IBD or IBS are not associated with intestinal inflammation, although potential harmful effects might be counteracted by anti-inflammatory components in the food matrix.

## 1. Introduction

The Maillard reaction is a biochemical reaction between proteins and reduced sugars that occurs during food processing, especially under conditions of heating. During this complex network of many thousands of individual non-enzymatic reactions, many different classes of Maillard reaction products (MRPs) are formed. Especially baking, grilling, and roasting of food products increases the MRP content of these foods. On one hand, this contributes to browning and organoleptic properties such as aroma, taste and texture, while on the other hand, MRPs are often reported as potentially harmful and, among others, are associated with impaired metabolic and gut health [1].

One of the endproducts of the Maillard reaction, namely the advanced glycation endproducts (AGEs), received considerable attention lately due to their potential negative effects on human health. The most well studied AGEs include Nε-(carboxymethyl)lysine (CML), Nε-(1-carboxyethyl)lysine (CEL) and methylglyoxal-derived hydroimidazolone-1 (MG-H1). In vitro and in vivo studies show that ingested AGEs can induce an inflammatory response [2,3,4,5] and affect microbial growth [5,6,7,8,9,10]. Furthermore, previous human studies showed that a diet high in dietary AGEs is associated with low-grade inflammation, endothelial dysfunction and insulin resistance [11]. In addition to AGEs, also their precursors may affect health. The dicarbonyls methylglyoxal (MGO), glyoxal (GO), and 3-deoxyglucosone (3-DG) are major precursors in the formation of AGEs. They are highly reactive intermediate metabolites and potent glycating agents, and have been associated with age-related diseases such as type 2 diabetes, cardiovascular diseases, and cancer [12,13]. Both pro-inflammatory [14] and anti-inflammatory effects [15,16] of the intake of dietary dicarbonyls have been reported.

Several in vitro and in vivo studies have shown that dietary dicarbonyls and AGEs are not completely digested and absorbed, with an absorption of 0.1–15% of consumed dicarbonyls [17,18] and 10–30% of consumed AGEs [19], depending on their chemical structure. The remaining dietary dicarbonyls and AGEs may therefore directly impact the mucosal layer of the small and large intestine [20,21,22], and/or may be metabolised by intestinal microbes [23]. Some animal and in vitro studies suggest that AGEs can infiltrate enterocytes and accumulate there [20,24].

It is now well known that a Western diet, being rich in processed food and thus in MRPs, is associated with common gastrointestinal diseases such as inflammatory bowel disease (IBD) and irritable bowel syndrome (IBS). IBD and IBS are both multifactorial and very heterogeneous entities in which diet likely plays a pathophysiological role [25,26]. IBD is a chronic inflammatory disease characterised by alternating sequences of active inflammation and remission [27,28]. IBS is characterised by abdominal pain and altered bowel habits, but in a subgroup of IBS a low-grade inflammation is reported [29]. Previous studies found an elevated expression of the receptor for AGEs, RAGE, in inflamed intestinal tissue from IBD patients [30,31,32], which may contribute to the production of pro-inflammatory cytokines and reactive oxygen species [33]. However, overall evidence on the role of dietary dicarbonyls and AGEs in IBD and IBS is limited.

Therefore, the aim of this study is to investigate the intake of dietary dicarbonyls and AGEs as part of the habitual diet in both IBD and IBS patients, and their association with intestinal inflammation. We hypothesise that dietary intake of dicarbonyls and AGEs is associated with intestinal inflammation in IBD and IBS.

## 2. Methods

### 2.1. Study Population

For this study, we used cross-sectional data on habitual dietary intake and clinical data from the IBD South Limburg (IBDSL) cohort and the Maastricht IBS (MIBS) cohort as described previously [34,35,36]. Prior to participation, all participants provided written informed consent.

#### 2.1.1. IBD South Limburg Cohort

Since 1991, the IBDSL cohort has been used to study IBD epidemiology and disease in the South Limburg area in the Netherlands [35]. This well-characterised population-based inception cohort comprised all newly diagnosed patients with ulcerative colitis (UC) and Crohn’s disease (CD) of at least 18 years old living in South Limburg at time of diagnosis. Diagnosis was done according to the Lennard-Jones criteria [37] and proven by endoscopic, radiological and/or histological findings. The IBDSL data warehouse was used to retrieve relevant demographical and clinical data [35]. The IBDSL cohort was approved by the medical research ethics committee of the Maastricht University Medical Center+ (MUMC+) (NL31636.068.10) and registered at the US National Library of Medicine (NCT02130349). The collection of data on habitual dietary intake was done as as part of a sub-study within the IBDSL cohort, also approved by the medical research ethics committee of the MUMC+ (NL42101.068.12) and registered at the US National Library of Medicine (NCT0176963).

#### 2.1.2. Maastricht IBS Cohort

Since 2009, the MIBS cohort has been used to study the phenotypical and genotypical characterisation of patients with IBS in the South Limburg area of the Netherlands. This cohort included IBS patients recruited via primary, secondary and tertiary care and from the general population that fulfilled the Rome III criteria and were at least 18 years [38]. Additionally, healthy controls (HC) in the same age category were included as described previously [36]. The medical research ethics committee of the MUMC+ approved the MIBS cohort (NL24160.068.08) and the study was registered at the US National Library of Medicine (NCT00775060).

### 2.2. Demographic and Clinical Data Collection

Standardised registration forms were used in both cohorts to collect demographic and clinical data, including sex, age, smoking status, body mass index (BMI), medication use and disease characteristics.

IBD disease phenotype at time of inclusion was defined by the Montreal classification, including age of onset, disease location, extent (for UC) and behaviour (for CD) [39]. Disease duration was also registered. The Simple Clinical Colitis Activity Index (SCCAI) [40] and Harvey Bradshaw Index (HBI) [41] were used as clinical activity indices for UC and CD, respectively. In line with clinical practice and previous studies [42,43], a flare was defined as: (1) presence of active disease based on endoscopy and/or radiological imaging, confirmed by a physician; (2) faecal calprotectin ≥ 250 μg/g; (3) faecal calprotectin ≥ 100 μg/g with at least a five-fold increase compared to the previous visit; (4) clinical symptoms indicative for active disease, or an increased SCCAI (≥3) or HBI (≥5) together with a dose escalation or initiation of a new drug; or (5) a dose escalation or initiation of a new drug along with C-Reactive Protein (CRP) ≥ 10 mg/l. Time since last flare was also recorded, and active disease at inclusion was defined as having a flare at inclusion, or during the three months prior to inclusion.

IBS subtypes were defined according to the Rome III criteria, i.e., diarrhoea (IBS-D) or constipation predominant (IBS-C), having a mixed stool pattern (IBS-M) or unspecified stool pattern (IBS-U) [38].

In both cohorts, intestinal inflammation was assessed by analyzing calprotectin levels in faecal samples. Participants collected these faecal samples at home, stored them in a fridge, and brought them to the hospital within 24 h after defecation. For the IBDSL cohort, samples were routinely analysed by the clinical chemistry department using a fluorescent enzyme immune assay (FEIA, Thermo Fisher Scientific, Waltham, MA, USA), whereas for the MIBS cohort samples were analysed using a commercial enzyme-linked immunosorbent assay (ELISA, Bühlmann Laboratories, Schönenbuch, Switzerland).

### 2.3. Dietary Data Collection

In both cohorts, the same self-administered food frequency questionnaire (FFQ) was used to assess the habitual dietary intake over the previous month. Frequency of consumption was scored per food product, and portion sizes were estimated using natural portions and commonly used household measures. These data were linked to the Dutch food composition table (NEVO online version 2010/2.0, National Institute for Public Health and the Environment (RIVM), Bilthoven, the Netherlands) to calculate the individual mean consumption of 45 nutrients and 148 food items. This FFQ was previously developed and validated by the division of Human Nutrition of Wageningen University [44,45]. Intake of nutritional supplements was recorded separately.

If the FFQ data were incomplete or considered implausible; i.e., defined as an overall intake for females <500 or >3500 kcal/day and for males <800 or >4000 kcal/day [46], or if the participant was on tube feeding, they were excluded from the analyses.

Foods and drinks were categorised in 25 food groups: (1) bread; (2) breakfast cereals; (3) cookies and bakery products; (4) potatoes, rice and pasta; (5) bread condiments; (6) vegetables and legumes; (7) fruits; (8) meat; (9) fish; (10) vegetarian and soy products; (11) milk and dairy products including cheese; (12) egg; (13) ready-made meals; (14) nuts and snacks; (15) fats and oils; (16) savoury sauces; (17) sweets and chocolate; (18) tea; (19) coffee; (20) soft drinks; (21) fruit juice; (22) vegetable juice; (23) beer; (24) wine; (25) liqueur.

Additionally, data were used to calculate the Dutch healthy diet index 2015 (DHD-2015) [47] and the Adapted Dietary Inflammatory Index (ADII) [48], as also described previously [34]. The DHD-2015, developed to assess the adherence to the Dutch healthy diet guidelines [49], was based on 13 components with a maximum score (indicating high adherence) of 130. The ADII was used to evaluate the inflammatory potential of the diet, with a more pro-inflammatory diet indicated by a higher (positive) score, whereas a more anti-inflammatory diet is indicated by a lower (negative) score.

#### Dietary Dicarbonyls & AGEs

The FFQ data were combined with available databases of dietary dicarbonyls MGO, GO and 3-DG [50], and dietary AGEs CML, CEL and MG-H1 [51]. For these databases the dicarbonyl and AGEs content of more than 200 foods and drinks were measured using ultra high-performance liquid chromatography tandem mass spectrometry (UHPLC-MS/MS) analysis (Acquity UPLC and Xexo TQ-MS, Waters, Milford, KS, USA) as described previously by Maasen et al. [50] and Scheijen et al. [51], respectively.

The average intake of each food product estimated by the FFQ (g/day) was multiplied by the amount of MGO, GO, 3-DG, CML, CEL and MG-H1 (mg/g) according to these databases, to calculate the daily dicarbonyl and AGE intake. For FFQ items that were not in the database, the average dicarbonyl or AGE concentration of comparable food products from the same food group was used as an estimate. Concentrations based on food items consumed were used to calculate the total intake of dietary dicarbonyls (MGO + GO + 3-DG) and dietary AGEs (CML + CEL + MG-H1). Furthermore, to correct for the impact of the amount of food consumed, the energy-adjusted intake (intake per 1000 kcal per day) was also calculated [46].

To calculate daily intake of dicarbonyls and AGEs for each food group, the concentration of a food product (mg/g) was multiplied by the individual’s daily intake of that food product (g/day), and subsequently all food products in a particular food group were summed. The relative contribution (as percentage of total intake) of each food group was determined.

### 2.4. Statistical Analyses

Statistical analyses were performed with IBM SPSS Statistics version 26.0 [52]. Data normality was confirmed by normal probability plots. For continuous parametric variables, baseline characteristics were presented as mean with corresponding standard deviation (SD), and differences between subgroups (i.e., IBD patients, IBS patients and HC) were assessed with analysis of variance (ANOVA) and post hoc Bonferroni correction. For categorical variables, baseline characteristics were presented as percentages, and differences between subgroups were assessed with the Chi-square test with Fisher exact when necessary.

To assess the association of dietary intake of dicarbonyls and AGEs with faecal calprotectin (as marker for intestinal inflammation), linear regression analysis was used, including the following parameters: age, sex, smoking, BMI, medication use, subtype (IBS) or phenotype (IBD), and for IBD patients additionally disease duration (in years) and age at diagnosis (defined by the Montreal classification). Analyses were performed for each subgroup (IBD, IBS and HC) separately and missing values were excluded listwise. A two-sided *p*-value < 0.05 was considered to be statistically significant.

In addition, clinically relevant cut-off points for faecal calprotectin [53] were used to define subgroups to further explore possible differences in dicarbonyls and AGEs intake with ANOVA and post hoc Bonferroni correction. Furthermore, correlations between dicarbonyls/AGEs and dietary indices (ADII and DHD-2015) were assessed using Spearman’s Rank-Order Correlation.

## 3. Results

### 3.1. Baseline Characteristics

FFQ data were available for 239 IBD patients, 274 IBS patients and 207 HC, of which 1 IBD patient, 13 IBS patients and 12 HC were excluded because of implausible high or low energy intake. This resulted in a final inclusion of 238 IBD patients, 261 IBS patients and 195 HC in the current study.

The IBD group comprised 82 UC (34.5%) and 156 CD (65.5%) patients. At time of inclusion, 61.5% of these patients (36.5% and 28.0%, respectively) were in remission. Among IBS patients, the main subtype was IBS-M (39.5%), followed by IBS-D (35.6%), IBS-C (21.5%), and IBS-U (3.4%).

Demographic and clinical data are shown in Table 1. The percentage of women was higher in the IBS group (74%) as compared to the IBD (52.9%, *p* < 0.001) and HC group (63.1%, *p* = 0.007). BMI was higher in IBD (25.5 ± 4.2 kg/m^2^) as well as IBS patients (25.0 ± 4.6 kg/m^2^) compared to HC (23.9 ± 3.8 kg/m^2^, with *p* < 0.001 and *p* = 0.021, respectively), and more active smokers were present among both IBD (20.4%, *p* < 0.001) and IBS patients (23.6%, *p* < 0.001) as compared to HCs (6.7%). The mean energy intake was lower in IBS patients (1939.6 ± 604.9 kcal) as compared to IBD patients (2180.0 ± 634.3 kcal, *p* < 0.001) and HC (2180.4 ± 622.9 kcal, *p* < 0.001). Further details on the intake of food items and specific nutrients were reported previously [34].

### 3.2. Intake of Dietary Dicarbonyls

Food groups with the highest contribution to the amount of MGO, GO and 3-DG were bread, cookies and bakery products, and vegetables and legumes. Furthermore, coffee was an important contributor for MGO and 3-DG, meat for MGO, fruit and ready-made meals for GO, and sweets and chocolate for 3-DG. For details, see Supplementary Figure S1. The main contributing products were comparable between subgroups.

The absolute intake of the dicarbonyls MGO, GO and 3-DG was lower in IBS as compared to IBD (all *p* < 0.05) and HC (all *p* < 0.05), but did not differ between IBD and HC (Table 2 and Supplementary Figure S2). When adjusted for the total energy intake (Supplementary Table S1), dietary GO levels were lower in IBD compared to IBS (*p* = 0.021) and HC (*p* = 0.040). The energy-adjusted intake of MGO and 3-DG was not significantly different between the groups.

### 3.3. Intake of Dietary AGEs

Food groups with the highest contribution to the amount of CML, CEL, MG-H1 were bread, cookies and bakery products and meat. Furthermore, dairy was an important contributor for CML, bread condiments for CEL, potatoes, rice and pasta for CML and MG-H1, and nuts and savoury snacks for CEL and MG-H1. For details, see Supplementary Figure S1. The main contributing products were comparable between subgroups.

The absolute intake of dietary AGEs CML, CEL and MG-H1 was lower in IBS compared to IBD (all *p* < 0.001) and HC (all *p* < 0.05), but was not significantly different between IBD and HC (Table 2 and Supplementary Figure S2). After adjustment for total energy intake (Supplementary Table S1), there were no longer any significant differences between the groups.

### 3.4. Intestinal Inflammation

Faecal calprotectin levels were available for 209 patients with IBD, 90 patients with IBS and 148 HC. Mean faecal calprotectin levels (Table 1) were significantly higher in IBD patients (197.3 ± 426.3 μg/g) versus IBS (64.6 ± 87.1 μg/g, *p* = 0.001) and HC (39.3 ± 63.6 μg/g, *p* < 0.001), but did not differ between IBS and HC (*p* > 0.999).

Based on the multivariable linear regression analysis (Table 3) faecal calprotectin was associated with GO in HC (β = −11.21, *p* = 0.045), but was not significantly associated with any of the other individual dietary dicarbonyls or AGEs, nor with the total amount of dicarbonyls or AGEs. The energy-adjusted intake of any of these compounds was also not associated with calprotectin (Supplementary Table S2).

As we only found a significant association in HCs, which was the group with the lowest calprotectin levels, we decided to also explore subgroups based on calprotectin levels rather than disease. Clinically relevant cut-offs for calprotectin were used to divide the total population in subgroups based on low (<15 μg/g), moderate (15–<50 μg/g) or high (50 μg/g or higher) faecal calprotectin levels (Supplemental Table S3). Assessment of significant differences in dietary dicarbonyls and AGEs intake between these calprotectin-based subgroups showed that the absolute intake of MGO was significantly higher in individuals with low calprotectin levels as compared to moderate calprotectin levels (*p* = 0.031). None of the other comparisons were significantly different between these subgroups.

### 3.5. Inflammatory Potential of Diet and Overall Diet Quality

We found food groups such as bread, vegetables and legumes, nuts, and fruits were among the food groups contributing most to the intake of dietary dicarbonyls and AGEs in all three groups. As these food groups are generally considered healthy because of their high content in components such as vitamins, minerals, and anti-oxidants, which might counteract the potential effects of dicarbonyls and AGEs, we also investigated whether the absolute intake of dicarbonyls and AGEs showed a correlation with the inflammatory potential of the diet, and/or with overall diet quality.

When evaluating the anti-inflammatory potential of the diet by the ADII (Supplementary Table S4), a higher absolute intake of MGO was correlated with a lower ADII in IBS (r = −0.169, *p* = 0.006) and HC (r = −0.195, *p* = 0.006). Furthermore, a higher intake of GO was correlated with a lower ADII in all groups (all *p* < 0.01). The intake of 3-DG was not significantly correlated with the ADII in either of the groups. A higher intake of CML was significantly correlated with a higher ADII (r = 0.216, *p* = 0.002) in HC only, while no correlations were found for CEL and MG-H1 in either of the groups. Furthermore, none of the summed intakes correlated significantly with the ADII in either of the subgroups.

With regard to overall diet quality (Supplementary Table S5), a higher absolute intake of the dicarbonyl GO and the AGE MG-H1, but not the others, were correlated with a higher DHD-2015 in all groups (all *p* < 0.05). The summed intake of dietary dicarbonyls was also not significantly associated with the DHD-2015 in either of the subgroups. Additionally, a higher intake of summed dietary AGEs but not total dicarbonyls was correlated with a higher DHD-2015 in IBS (r = 0.141 and *p* = 0.022) and HC (r = 0.178 and *p* = 0.013).

## 4. Discussion

To our best knowledge, this is the first study investigating the intake of dietary dicarbonyls and AGEs in IBD and IBS patients. We found that the absolute intake of both was lower in patients with IBS as compared to IBD and HC, but not after adjustment for energy-intake. The intake of dietary dicarbonyls and AGEs was not significantly associated with faecal calprotectin in IBD and IBS patients, apart from a higher MGO intake in individuals with low as compared to moderate calprotectin levels, indicating a potential protective effect. Furthermore, a higher intake of dicarbonyls and AGEs was not associated with a lower diet quality or higher inflammatory potential of the diet, except for a significant positive correlation between CML intake and the ADII in HC.

In the current study, overall intake of dicarbonyls and AGEs was not higher in IBD and IBS as compared to controls and intakes were largely in line with previous findings in other Dutch cohorts including healthy individuals, and those at increased risk of or with type 2 diabetes [54,55,56]. In contrast, even lower absolute but not energy-adjusted levels of all dicarbonyls and AGEs were found in IBS, and lower energy adjusted concentration, but not absolute intake of GO was found in IBD patients.

Although several studies found an association of dietary intake of MRPs with plasma and tissue levels of dicarbonyls [17] and AGEs [57,58], there is also evidence that AGEs are only partially digested and absorbed [18,59], indicating that a large proportion reaches the colon. Therefore, MRPs may have a local inflammatory effect in the intestine. However, we found no association between higher intake levels and higher faecal calprotectin levels in either of the subgroups studied. On the contrary, we found a higher absolute MGO in individuals with low as compared to moderate calprotectin levels. This is in line with a recent study that found a higher habitual intake of MGO to be associated with less low-grade inflammation as measured in plasma [16]. Nonetheless, no differences were found when comparing the other dietary dicarbonyls and AGEs in those with low, moderate or high calprotectin levels.

Furthermore, we found that a higher intake of dicarbonyls and AGEs was generally associated with a better diet quality and a more anti-inflammatory diet. Thereby, in the current study, we find no evidence for a higher intake of dicarbonyls and AGEs being associated with intestinal inflammation in IBD or IBS patients as compared to HC, nor for an association with diet.

In line with previous studies [50,60,61], we found the main food products contributing to the intake of dietary dicarbonyls and AGEs in IBD and IBS patients were not only processed foods such as cookies and bakery products, sweets/chocolate and savoury snacks, but also products generally considered to be healthy such as bread, vegetables, legumes, fruit, potatoes, rice and pasta, and coffee. This food matrix is important to consider when investigating the health effects of any food components, as they contain anti-oxidants, fibres and micronutrients that may protect against the dicarbonyls and AGEs [62,63,64]. Therefore, we cannot rule out that any potential detrimental health effect from the dicarbonyls and AGEs are counteracted by the anti-inflammatory components of these healthy foods. It should also be emphasised that some studies even indicate a hormetic effect of dicarbonyls [65,66] and AGEs [67], and animal studies showing harmful effects are mostly based on supraphysiologic levels of intake [3,68].

Additionally, the food matrix should be considered for its effect on digestion and absorption. A study using the standardised TNO in vitro gastrointestinal digestion model (TIM-1), showed that the protein-bound form of AGEs can survive gastric and small intestinal digestive secretions, and stays intact during upper GI tract passage [59]. Additionally, in vitro evidence indicates that dietary dicarbonyls reach the colon largely unaltered by digestion [69]. With these undigested MRPs being present in the GI tract together with proteins from the food matrix or the intestinal environment, the Maillard reaction can also occur endogenously in the GI tract, involving a bidirectional interaction with the intestinal microbiome. Several animal studies have shown that a heat-treated chow diet, high in dietary AGEs, can lead to the gut microbiota composition perturbations [5,6,8,68,70,71]. On the other hand, studies with mice on a lactose or fructo-oligosaccharide-diet resulted in an increased colonic epithelial RAGE expression, increased mucosal mast cells numbers and activity, abdominal hypersensitivity [72], and a dysregulation of the colonic mucus barrier [73]. As this was accompanied by increased CML levels in the colonic epithelium, and was prevented by co-treatment with pyridoxamine, a known anti-glycation agent, this points towards microbial involvement in glycation processes [73]. As the intestinal microbiota displays large inter-individual variation and moreover differences in composition have been shown in IBD and IBS as compared to controls [74], further studies are needed to study the impact of the endogenous dicarbonyl and AGEs generation and the involvement of the individual’s microbiota composition and activity.

The databases used in our study were both based on UHPLC-MS/MS analysis, which is considered to be the best analytical method to quantify dicarbonyls [50] and AGEs [51] in food. Nevertheless, it is important to mention the limitation that only six components, i.e. three dicarbonyls and three AGEs, were included in these databases, whereas foods contain many more MRPs. Furthermore, an important limitation from our FFQ is that it does not include detailed information about food preparation methods for all food items. Several studies showed cooking techniques and heating are fundamental in the formation of MRPs [50,51,55,61,75]. However, the effect of this missing information is considered to be limited because the databases contained mostly uncooked or pre-processed foods, and cooked foods were prepared according to the manufacturer’s label or using the most common preparation technique.

## 5. Conclusions

Dietary intake of dicarbonyls and AGEs was not higher in IBD and IBS patients as compared to healthy controls, when adjusted for overall energy intake. Furthermore, in this study we found no leads that the concentrations of dicarbonyls and AGEs generally present in the diet of Dutch patients with IBD or IBS are associated with intestinal inflammation. However, we cannot rule out potential harmful effects might be counteracted by anti-inflammatory components in the food matrix, so further studies investigating this are needed.

## Figures and Tables

**Table 1:** Baseline characteristics.

	IBD Patients (*n* = 238)	IBS Patients (*n* = 261)	HC (*n* = 195)	*p*-Value
Age (years)	45.7 ± 14.8	43.3 ± 17.0	44.4 ± 18.9	0.285
Sex				<0.001
Male	47.1%	25.3%	36.9%	
Female	52.9%	74.7%	63.1%	
BMI (kg/m^2^) *	25.5 ± 4.2	25.0 ± 4.6	23.9 ± 3.8	<0.001
Smoking **				<0.001
Active smoker	20.4%	23.6%	6.7%	
Former smoker	41.7%	24.4%	31.8%	
Never smoker	37.9%	52.0%	61.5%	
IBD Phenotype				
Ulcerative colitis	34.5%	n/a	n/a	n/a
Crohn’s disease	65.5%	n/a	n/a	n/a
Age of onset **				
A1—below 17 years old	5.9%	n/a	n/a	n/a
A2—17–40 years old	64.0%	n/a	n/a	n/a
A3—above 40 years old	30.1%	n/a	n/a	n/a
Extent of ulcerative colitis (UC) at inclusion **				
E1—ulcerative proctitis	11.1%	n/a	n/a	n/a
E2—left sided UC (distal UC)	39.5%	n/a	n/a	n/a
E3—extensive UC (pancolitis)	49.4%	n/a	n/a	n/a
Behaviour of Crohn’s disease at inclusion				
B1—non-stricturing, non-penetrating	57.1%	n/a	n/a	n/a
B2—stricturing	17.9%	n/a	n/a	n/a
B3—penetrating	25.0%	n/a	n/a	n/a
Location of Crohn’s disease at inclusion				
L1—ileal	23.7%	n/a	n/a	n/a
L2—colonic	16.7%	n/a	n/a	n/a
L3—ileocolonic	59.6%	n/a	n/a	n/a
L4—upper-GI modifier	10.3%	n/a	n/a	n/a
Disease activity at inclusion				
Active disease	34.9%	n/a	n/a	n/a
Remission	61.5%	n/a	n/a	n/a
Disease duration (years) **	11.5 ± 10.1	n/a	n/a	n/a
Time to last flare (months)	37.7 ± 67.7	n/a	n/a	n/a
Bowel resection at inclusion				
Yes	23.1%	n/a	n/a	n/a
No	76.9%	n/a	n/a	n/a
Symptom score *				
Simple Clinical Colitis Activity Index	1.2 ± 1.8	n/a	n/a	n/a
Harvey Bradshaw Index	2.9 ± 3.4	n/a	n/a	n/a
IBS Subtype				
Constipation predominant IBS	n/a	21.5%	n/a	n/a
Diarrhoea predominant IBS	n/a	35.6%	n/a	n/a
Mixed stool pattern IBS	n/a	39.5%	n/a	n/a
Unspecified subtype IBS	n/a	3.4%	n/a	n/a
Faecal calprotectin (μg/g) ***	197.3 ± 426.3	64.4 ± 87.1	39.3 ± 63.6	<0.001
Medication ****				
No medication	14.3%	26.8%	52.8%	<0.001
5-ASA, local immunosuppressants, or local corticosteroids	17.6%	n/a	n/a	n/a
Systemic corticosteroids	0.4%	n/a	n/a	n/a
Immunomodulators	22.7%	n/a	n/a	n/a
Biologicals	45.0%	n/a	n/a	n/a
PPIs	n/a	20.7%	3.1%	<0.001
NSAIDs	n/a	24.9%	20.0%	0.217
Laxatives	n/a	18.4%	0.0%	n/a
Spasmolytic drugs	n/a	14.2%	0.0%	n/a
Antihypertensive drugs	n/a	15.3%	13.3%	0.550
Statins	n/a	10.0%	7.7%	0.402
Antidepressant drugs	n/a	10.0%	3.6%	0.009
Energy intake (kcal/day)	2180.0 ± 634.4	1939.6 ± 604.9	2180.4 ± 622.9	<0.001

IBD = inflammatory bowel disease; IBS = irritable bowel syndrome; HC = healthy controls; BMI = body mass index; 5-ASA = 5-aminosalicylic Acid; PPIs = proton pump inhibitors; NSAIDs = non-steroidal anti-inflammatory drugs; n/a = not applicable or not available. * Missing data from max. 25 participants per subgroup. ** Missing data from max. 3 participants per subgroup. *** Missing data from 29 IBD patients, 171 IBS patients and 47 HC. **** Missing data from 4 IBS patients. Medication for IBD patients was classified as the highest category of use. For IBS, only medication frequently used in IBS were presented. Other medication included prokinetics, anti-diarrhoeal drugs, oral contraceptives, antipsychotic drugs and antibiotics. Continuous data is expressed as mean ± standard deviation (SD), and differences between IBD, IBS and HC were tested with analysis of variance (ANOVA) and post hoc Bonferroni correction. Categorical data is expressed as percentages of total group (IBD, IBS or HC), and differences between IBD, IBS and HC were assessed with the Chi-square test with Fisher for categorical data.

**Table 2:** Absolute dietary intake of individual dicarbonyls and advanced glycation endproducts.

Absolute Intake (mg/Day, Mean ± SD)	IBD Patients (*n* = 238)	IBS Patients (*n* = 261)	HC (*n* = 195)	*p*-Value
MGO	4.04 ± 1.59	3.53 ± 1.46	3.94 ± 1.45	<0.001
GO	3.32 ± 1.04	3.09 ± 0.96	3.49 ± 1.06	<0.001
3-DG	15.55 ± 6.44	13.76 ± 5.85	15.83 ± 5.75	<0.001
Dicarbonyls	22.91 ± 8.23	20.38 ± 7.50	23.26 ± 7.54	<0.001
CML	3.35 ± 1.16	2.91 ± 1.07	3.27 ± 1.16	<0.001
CEL	2.70 ± 0.93	2.40 ± 0.83	2.64 ± 0.94	<0.001
MG-H1	22.61 ± 7.97	19.97 ± 7.32	23.06 ± 7.84	<0.001
AGEs	28.67 ± 9.79	25.28 ± 9.02	28.97 ± 9.69	<0.001

IBD = inflammatory bowel disease; IBS = irritable bowel syndrome; HC = healthy controls; SD = standard deviation; MGO = methylglyoxal; GO = glyoxal; 3-DG = 3-deoxyglucosone; CML = Nε-(carboxymethyl)lysine; CEL = Nε-(1-carboxyethyl)lysine; MG-H1 = methylglyoxal-derived hydroimidazolone-1; AGEs = advanced glycation endproducts. The differences between IBD, IBS and HC were assessed with analysis of variance (ANOVA) and post hoc Bonferroni correction.

**Table 3:** Multivariable linear regression of absolute dietary intake of dicarbonyls and advanced glycation endproducts with faecal calprotectin.

	IBD Patients (*n* = 209)			IBS Patients (*n* = 90)			HC (*n* = 148)		
	β	95% CI	*p*-Value	β	95% CI	*p*-Value	β	95% CI	*p*-Value
MGO	−19.01	−48.57; 10.59	0.206	6.44	−7.25; 20.12	0.352	−4.77	−13.71; 4.18	0.294
GO	−20.80	−65.04; 23.45	0.355	−0.50	−21.42; 20.41	0.962	−11.21	−22.17; −0.25	0.045
3-DG	−1.28	−8.61; 6.04	0.730	−1.38	−4.59; 1.82	0.393	−0.86	−2.77; 1.05	0.374
Dicarbonyls	−1.83	−7.54; 3.88	0.528	−0.67	−3.25; 1.91	0.606	−0.09	−2.33; 0.63	0.258
CML	−35.48	−75.31; 4.35	0.080	−0.87	−19.78; 17.44	0.925	−1.99	−12.02; 8.04	0.696
CEL	−30.29	−81.11; 20.54	0.241	−1.97	−24.03; 20.09	0.859	−0.87	−13.26; 11.54	0.891
MG-H1	−1.67	−7.74; 4.42	0.590	−0.22	−2.70; 2.27	0.863	−0.51	−1.98; 0.96	0.491
AGEs	−1.91	−6.84; 3.02	0.445	−0.17	−2.21; 1.86	0.867	−0.37	−1.56; 0.82	0.538

IBD = inflammatory bowel disease; IBS = irritable bowel syndrome; HC = healthy controls; β = regression coefficient; 95% CI = 95% confidence interval; MGO = methylglyoxal; GO = glyoxal; 3-DG = 3-deoxyglucosone; CML = Nε-(carboxymethyl)lysine; CEL = Nε-(1-carboxyethyl)lysine; MG-H1 = methylglyoxal-derived hydroimidazolone-1; AGEs = advanced glycation endproducts. Faecal calprotectin was measured in μg/g. Analyses were performed using multivariable linear regression with faecal calprotectin levels as dependent variable, and were corrected for: age, sex, smoking, BMI, disease specific medication (all subgroups), plus phenotype, disease duration (years) and age of onset according to the Montreal classification for IBD, or plus subtype for IBS.

## Data Availability

The data presented in this study are available upon reasonable request from the corresponding author.

## Supplementary Materials

The following supporting information can be downloaded at: https://www.mdpi.com/article/10.3390/nu15010083/s1, Supplementary Figure S1. Main contributing food group (%) for absolute dietary intake of individual dicarbonyls and dietary advanced glycation endproducts (for inflammatory bowel disease, irritable bowel syndrome and healthy controls combined); Supplementary Figure S2. Stacked bar chart of sum scores for absolute dietary intake of dicarbonyls methylglyoxal (MGO), glyoxal (GO) and 3-deoxyglucosone (3-DG), and advanced glycation endproducts (AGEs) Nε-(carboxymethyl)lysine (CML), Nε-(1-carboxyethyl)lysine (CEL) and methylglyoxal-derived hydroimidazolone-1 (MG-H1) for inflammatory bowel disease (IBD) patients, irritable bowel syndrome (IBS) patients, and healthy controls (HC); Supplementary Table S1. Energy-adjusted dietary intake of dicarbonyls and advanced glycation endproducts; Supplementary Table S2. Multivariable linear regression of energy-adjusted dietary intake of dicarbonyls and advanced glycation endproducts with faecal calprotectin; Supplementary Table S3. Comparison of absolute dietary intake of dicarbonyls and advanced glycation endproducts (individual values and sum scores) for subgroups based on clinically relevant cut-off points; Supplementary Table S4. Spearman’s Rank-Order Correlation of dietary intake of dicarbonyls and advanced glycation endproducts with the Adapted Dietary Inflammatory Index; Supplementary Table S5. Spearman’s Rank-Order Correlation of dietary intake of dicarbonyls and advanced glycation endproducts with the Dutch Healthy Diet Index 2015.

## Abbreviations

3-DG	3-deoxyglucosone
ADII	Adapted Dietary Inflammatory Index
AGEs	advanced glycation endproducts
ANOVA	analysis of variance
BMI	body mass index
CD	Crohn’s disease
CEL	Nε-(1-carboxyethyl)lysine
CML	Nε-(carboxymethyl)lysine
CRP	C-Reactive Protein
DHD-2015	Dutch Healthy Diet Index 2015
ELISA	enzyme-linked immunosorbent assay
FEIA	fluorescent enzyme immune assay
FFQ	food frequency questionnaire
GI	gastrointestinal
GO	glyoxal
HBI	Harvey Bradshaw Index
HC	healthy controls
IBD	inflammatory bowel disease
IBDSL	IBD South Limburg
IBS	irritable bowel syndrome
IBS-C	constipation predominant IBS
IBS-D	diarrhoea predominant IBS
IBS-M	mixed stool pattern IBS
IBS-U	unspecified subtype IBS
MIBS	Maastricht IBS
MG-H1	methylglyoxal-derived hydroimidazolone-1
MGO	methylglyoxal
MRPs	Maillard reaction products
MUMC+	Maastricht University Medical Center+
RAGE	receptor for advanced glycation endproducts
SCCAI	Simple Clinical Colitis Activity Index
TIM-1	TNO in vitro gastrointestinal digestion model
SD	standard deviation
UC	ulcerative colitis
UHPLC-MS/MS	ultra high-performance liquid chromatography tandem mass spectrometry
