# Supplementary material for: The Intake of Dicarbonyls and Advanced Glycation Endproducts as Part of the Habitual Diet Is Not Associated with Intestinal Inflammation in Inflammatory Bowel Disease and Irritable Bowel Syndrome Patients
Source: Nutrients. 2022 Dec 24;15(1):83. doi: 10.3390/nu15010083 (PMC9824683; doi:10.3390/nu15010083)
Supplement: Supplementary file 1 [file nutrients-15-00083-s001.zip › nutrients-2072713-supplementary.pdf]

Supplementary File

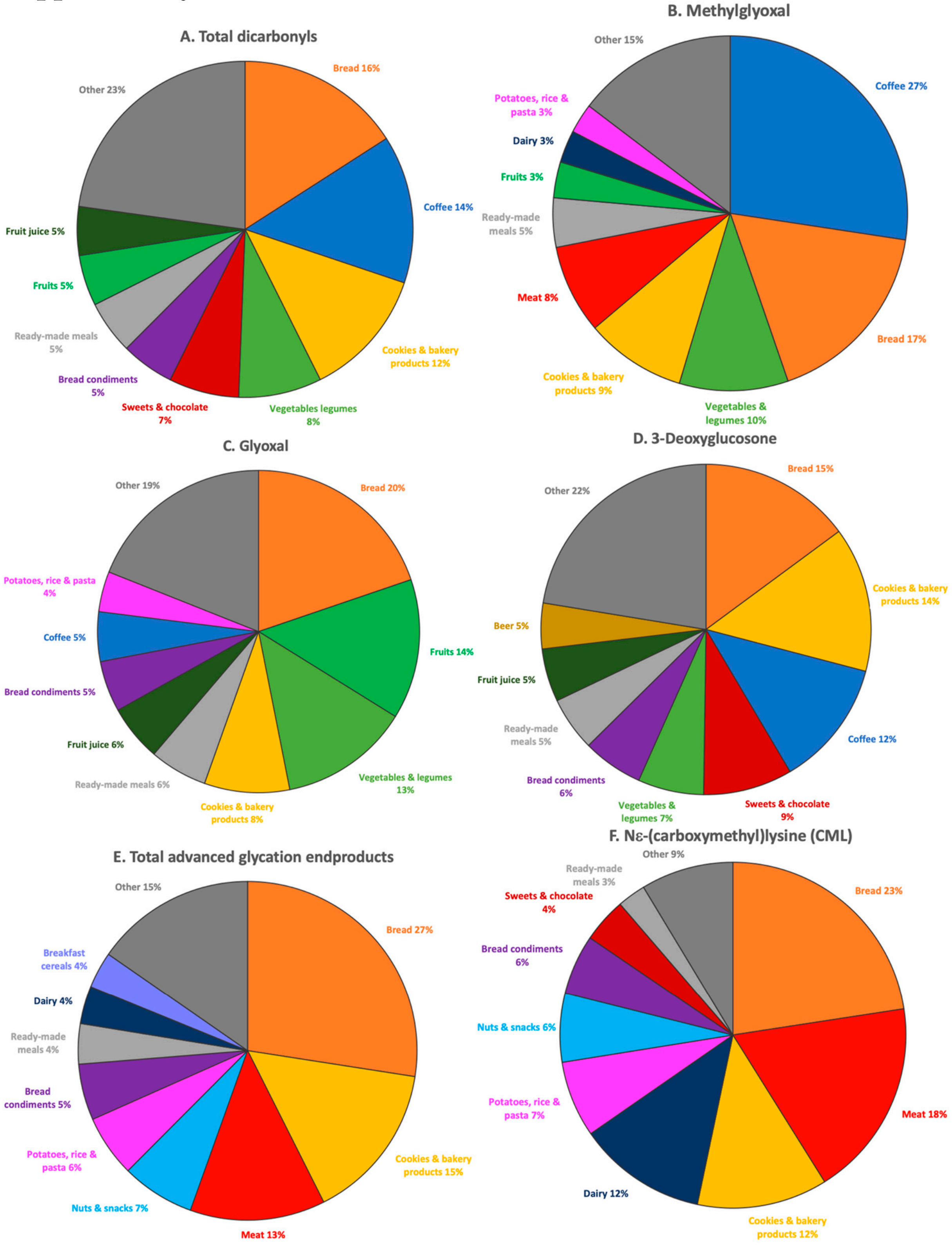

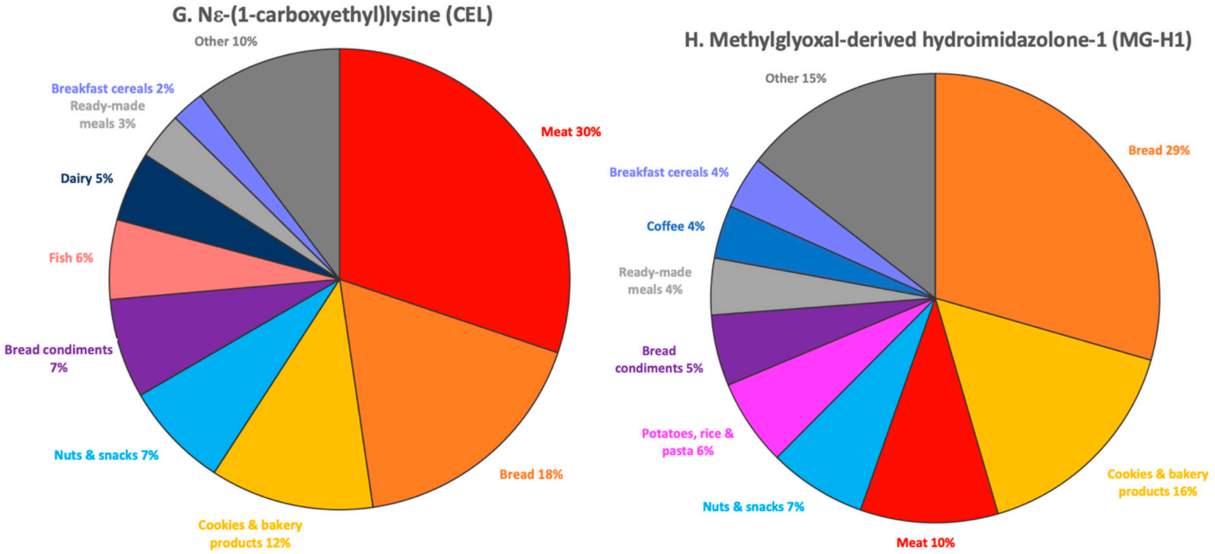

Supplemental Figure S1. Main contributing food group (%) for absolute dietary intake of individual dicarbonyls and dietary advanced glycation endproducts (for inflammatory bowel disease, irritable bowel syndrome and healthy controls combined).

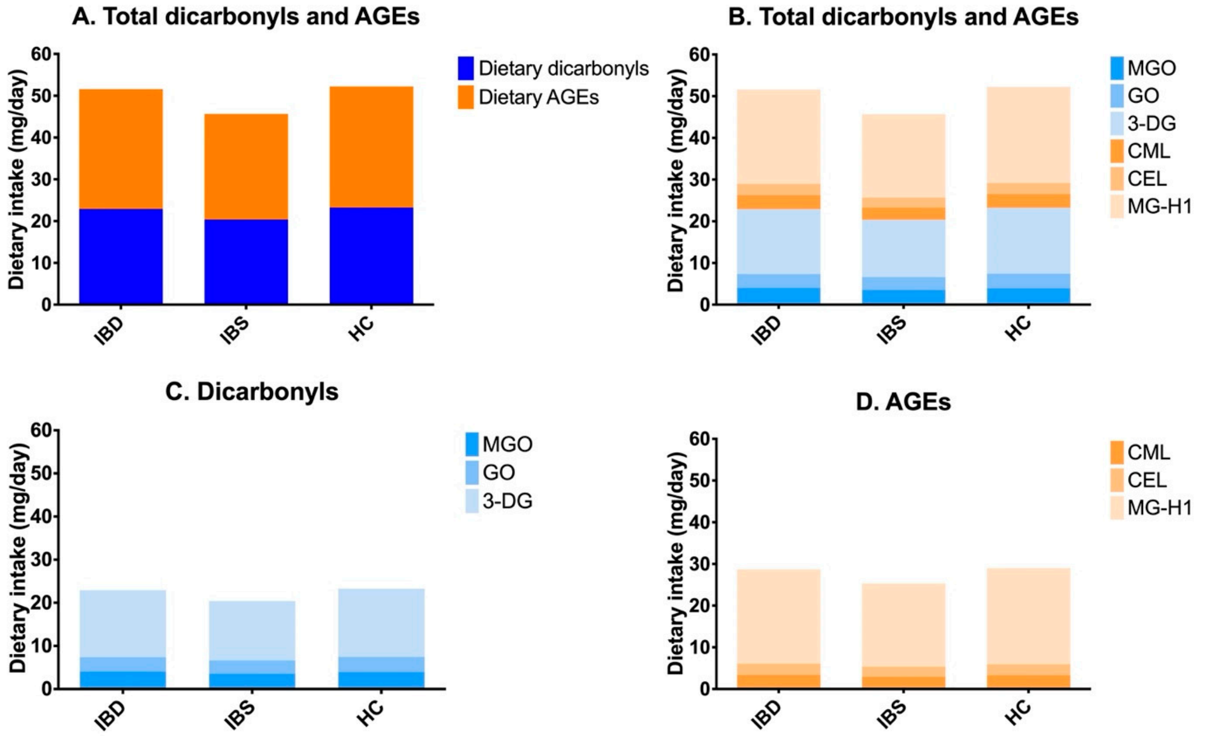

Supplemental Figure S2. Stacked bar chart of sum scores for absolute dietary intake of dicarbonyls methylglyoxal (MGO), glyoxal (GO) and 3-deoxyglucosone (3-DG), and advanced glycation endproducts (AGEs) Nε-(carboxymethyl)lysine (CML), Nε-(1-carboxyethyl)lysine (CEL) and methylglyoxal-derived hydroimidazolone-1 (MG-H1) for inflammatory bowel disease (IBD) patients, irritable bowel syndrome (IBS) patients, and healthy controls (HC).

Supplemental Table S1. Energy-adjusted dietary intake of dicarbonyls and advanced glycation endproducts.

Energy-adjusted intake (mg/day, Mean ± SD)	IBD patients (*n* = 238)	IBS patients (*n* = 261)	HC (*n* = 195)	*p*-Value
MGO	1.91 ± 0.75	1.87 ± 0.72	1.85 ± 0.61	0.636
GO	1.55 ± 0.32	1.62 ± 0.35	1.62 ± 0.30	0.011
3-DG	7.23 ± 2.44	7.16 ± 2.22	7.32 ± 1.86	0.756
Dicarbonyls	10.69 ± 3.07	10.66 ± 2.78	10.80 ± 2.35	0.861
CML	1.54 ± 0.30	1.50 ± 0.31	1.50 ± 0.29	0.364
CEL	1.24 ± 0.24	1.25 ± 0.28	1.21 ± 0.26	0.259
MG-H1	10.38 ± 2.02	10.38 ± 2.39	10.63 ± 2.07	0.401
AGEs	13.16 ± 2.32	13.14 ± 2.77	13.35 ± 2.44	0.655

IBD = inflammatory bowel disease; IBS = irritable bowel syndrome; HC = healthy controls; SD = standard deviation; MGO = methylglyoxal; GO = glyoxal; 3-DG = 3-deoxyglucosone; CML = Nε-(carboxymethyl)lysine; CEL = Nε-(1-carboxyethyl)lysine; MG-H1 = methylglyoxal-derived hydroimidazolone-1; AGEs = advanced glycation endproducts. The differences between IBD, IBS and HC were tested with analysis of variance (ANOVA) and post-hoc Bonferroni correction.

Supplemental Table S2. Multivariable linear regression of energy-adjusted dietary intake of dicarbonyls and advanced glycation endproducts with faecal calprotectin.

	IBD patients (*n* = 209)			IBS patients (*n* = 90)			HC (*n* = 148)		
	β	95% CI	*p*-Value	β	95% CI	*p*-Value	β	95% CI	*p*-Value
MGO	6.82	-52.32; 65.97	0.820	9.39	-19.96; 38.74	0.526	-3.27	-24.74; 18.21	0.764
GO	34.14	-106.18; 174.45	0.632	-15.08	-59.44; 29.27	0.500	-28.23	-64.61; 8.16	0.127
3-DG	9.52	-9.84; 28.89	0.333	-5.77	-13.70; 2.18	0.151	0.72	-5.41; 6.85	0.816
Dicarbonyls	6.58	-8.47; 21.63	0.389	-3.65	-10.11; 2.81	0.264	-0.22	-5.15; 4.72	0.932
CML	-68.92	-214.78; 76.94	0.352	-30.91	-89.88; 28.06	0.300	26.03	-12.94; 64.99	0.189
CEL	-18.05	-197.98; 161.88	0.843	-44.10	-108.02; 19.82	0.173	37.20	-6.25; 80.65	0.093
MG-H1	6.84	-14.41; 28.08	0.526	-4.20	-10.28; 1.89	0.173	1.09	-4.36; 6.54	0.693
AGEs	3.86	-14.59; 22.30	0.680	-3.72	-9.00; 1.56	0.165	1.56	-3.06; 6.18	0.506

IBD = inflammatory bowel disease; IBS = irritable bowel syndrome; HC = healthy controls; β = regression coefficient; 95% CI = 95% confidence interval; MGO = methylglyoxal; GO = glyoxal; 3-DG = 3-deoxyglucosone; CML = Nε-(carboxymethyl)lysine; CEL = Nε-(1-carboxyethyl)lysine; MG-H1 = methylglyoxal-derived hydroimidazolone-1; AGEs = advanced glycation endproducts.

Faecal calprotectin was measured in μg/g.

Analyses were performed using multivariable linear regression with faecal calprotectin levels as dependent variable, and were corrected for: age, sex, smoking, BMI, disease specific medication (all subgroups), plus phenotype, disease duration (years) and age of onset according to the Montreal classification for IBD, or plus subtype for IBS

Supplemental Table S3. Comparison of absolute dietary intake of dicarbonyls and advanced glycation endproducts (individual values and sum scores) for subgroups based on clinically relevant cut-off points.

**Absolute intake (mg/day, Mean ± SD)**	**< 15 µg/g (*n* = 153)**	**15 – <50 µg/g (*n* = 136)**	**≥ 50 µg/g (*n* = 158)**	***p*-Value**
MGO	4.20 ± 1.66	3.75 ± 1.36	3.86 ± 1.52	0.031*
GO	3.48 ± 1.01	3.34 ± 0.99	3.28 ± 1.08	0.220
3-DG	15.50 ± 6.09	15.89 ± 6.36	15.11 ± 5.80	0.547
Dicarbonyls	23.18 ± 8.05	22.97 ± 7.96	22.26 ± 7.51	0.551
CML	3.27 ± 1.07	3.36 ± 1.23	3.18 ± 1.16	0.436
CEL	2.58 ± 0.88	2.71 ± 0.95	2.64 ± 1.01	0.557
MG-H1	22.04 ± 7.42	22.52 ± 7.69	22.76 ± 8.73	0.720
AGEs	27.89 ± 9.11	28.58 ± 9.60	28.58 ± 10.71	0.777

IBD = inflammatory bowel disease; IBS = irritable bowel syndrome; HC = healthy controls; SD = standard deviation; MGO = methylglyoxal; GO = glyoxal; 3-DG = 3-deoxyglucosone; CML = Nε-(carboxymethyl)lysine; CEL = Nε-(1-carboxyethyl)lysine; MG-H1 = methylglyoxal-derived hydroimidazolone-1; AGEs = advanced glycation endproducts. The differences between IBD, IBS and HC were tested with analysis of variance (ANOVA) and post-hoc Bonferroni correction.

* Post-hoc Bonferroni showed p=0.036 for <15 µg/g vs. 15 – <50 µg/g, other comparisons not significant.

Supplemental Table S4. Spearman's Rank-Order Correlation of dietary intake of dicarbonyls and advanced glycation endproducts with the Adapted Dietary Inflammatory Index.

	**IBD (*n* = 238)**		**IBS (*n* = 261)**		**HC (*n* = 195)**	
	**r**	***p*-Value**	**r**	***p*-Value**	**r**	***p*-Value**
MGO	-0.115	0.075	-0.169	0.006	-0.195	0.006
GO	-0.244	<0.001	-0.277	<0.001	-0.197	0.006
3-DG	0.034	0.600	0.019	0.760	0.091	0.205
Dicarbonyls	-0.025	0.699	-0.049	0.434	0.001	0.992
CML	0.111	0.089	0.101	0.102	0.216	0.002
CEL	-0.005	0.934	0.024	0.695	0.131	0.067
MG-H1	-0.044	0.496	0.017	0.783	0.097	0.178
AGEs	-0.021	0.747	0.029	0.645	0.116	0.105

IBD = inflammatory bowel disease; IBS = irritable bowel syndrome; HC = healthy controls; r = correlation coefficient; MGO = methylglyoxal; GO = glyoxal; 3-DG = 3-deoxyglucosone; CML = Nε-(carboxymethyl)lysine; CEL = Nε-(1-carboxyethyl)lysine; MG-H1 = methylglyoxal-derived hydroimidazolone-1; AGEs = advanced glycation endproducts.

Supplemental Table S5. Spearman's Rank-Order Correlation of dietary intake of dicarbonyls and advanced glycation endproducts with the Dutch Healthy Diet Index 2015.

	IBD (*n* = 238)		IBS (*n* = 261)		HC (*n* = 195)	
	**r**	***p*-Value**	**r**	***p*-Value**	**r**	***p*-Value**
MGO	-0.008	0.899	0.089	0.150	0.140	0.052
GO	0.202	0.002	0.166	0.007	0.480	<0.001
3-DG	-0.018	0.787	-0.070	0.261	0.035	0.627
Dicarbonyls	0.014	0.835	-0.020	0.743	0.091	0.206
CML	-0.040	0.543	0.022	0.725	0.078	0.281
CEL	-0.038	0.561	-0.011	0.856	0.066	0.359
MG-H1	0.129	0.047	0.176	0.004	0.204	0.004
AGEs	0.093	0.153	0.141	0.022	0.178	0.013

IBD = inflammatory bowel disease; IBS = irritable bowel syndrome; HC = healthy controls; r = correlation coefficient; MGO = methylglyoxal; GO = glyoxal; 3-DG = 3-deoxyglucosone; CML = Nε-(carboxymethyl)lysine; CEL = Nε-(1-carboxyethyl)lysine; MG-H1 = methylglyoxal-derived hydroimidazolone-1; AGEs = advanced glycation endproducts.
